# Radiographic Structural Damage Is Worse in the Dominant than the Non-Dominant Hand in Individuals with Early Rheumatoid Arthritis

**DOI:** 10.1371/journal.pone.0135409

**Published:** 2015-08-06

**Authors:** Jung Hee Koh, Seung Min Jung, Jennifer Jooha Lee, Kwi Young Kang, Seung-Ki Kwok, Sung-Hwan Park, Ji Hyeon Ju

**Affiliations:** Division of Rheumatology, Department of Internal Medicine, College of Medicine, The Catholic University of Korea, Seoul, South Korea; University of Hull, UNITED KINGDOM

## Abstract

**Objective:**

The relationship between mechanical stress and radiographic progression in rheumatoid arthritis (RA) is unclear. The assumption is that mechanical stress is greater in the dominant hand. Therefore, the aim of the present study was to compare the presence and progression of erosions and joint space narrowing (JSN) in the dominant and non-dominant hand.

**Methods:**

Data from 194 patients recently diagnosed with seropositive RA, and with hand radiographs taken at the time of diagnosis and at 2-year follow-up, were analyzed retrospectively. Radiographs were scored using the van der Heijde-modified Sharp Score (HSS) method. Each joint group within each hand was rated separately by two independent examiners in a double-blinded manner.

**Results:**

One hundred and ninety-four patients were enrolled (80% female, 88% positive rheumatoid factor, 92% positive anti-citrullinated protein antibody, and 95.4% right-handed). The baseline, follow-up erosion and JSN HSS were significantly higher in the dominant hand than in the non-dominant hand. The annual rate of radiographic progression was also higher in the dominant hand. The erosive progression in the wrist joints varied significantly according to handedness, but the erosion in the proximal interphalangeal joints and metacarpophalangeal joints was similar in both hands. The radiographic progression was associated with the dominant hand, an abnormal baseline C-reactive protein level, and joint damage at baseline. There was no significant difference in bone mineral density between the right and left hands.

**Conclusion:**

Radiological damage was worse and progressed faster in the dominant hand, suggesting that mechanical stress is associated with radiographic joint damage in early and active RA.

## Introduction

Simultaneous and symmetrical joint involvement is a hallmark of rheumatoid arthritis (RA) and was included as a diagnostic criterion defined by the American College of Rheumatology in 1987 [1]. The levels of radiographic damage in the dominant and non-dominant hand both at a specific time point and at follow-up are highly correlated [2]. Asymmetric hand involvement is often observed in patients with osteoarthritis (OA) and the majority of individuals with asymmetric hand OA presented in their dominant hand [3, 4]. The cartilage degeneration in OA can be correlated with abnormal or excessive articular contact stress [5]. Thus handedness [4] and occupational load [6, 7] are considered as possible causes of those asymmetric hand OA. Similarly, studies show that RA patients with hemiplegia or paralysis tend to suffer asymmetrical joint damage [8–10], suggesting that intra-articular mechanical stress is an important factor contributing to radiographic progression of RA. However, few studies have examined the severity and progression of joint damage in the right and left hands simultaneously.

Radiographic joint damage occurs early and progresses after RA diagnosis [11, 12] and has a significant impact on physical function [13, 14]. In general, joint inflammation such as joint swelling and increased acute phase reactant in RA leads to structural damage, which manifests as erosions and joint space narrowing (JSN) [15]. The latter is also a feature of osteoarthritis (OA) of the hand. Although joint erosion and JSN are included in most image scoring systems for RA, less focus is placed on erosion and JSN as separate entities.

Therefore, to gain insight into the relationship between mechanical stress and radiographic progression of RA in the hand, we separately analyzed joint erosions and JSN on radiographs taken of the dominant and non-dominant hand of early RA patients both at baseline and at 2-year follow-up. As periarticular osteoporosis is the earliest radiological changes in RA [16, 17], we also analyzed hand bone mineral density (BMD) data to postulate association between mechanical stress and BMD as early changes before radiographic joint destruction. If the frequent use of a hand facilitates joint damage, such information would be useful for the management of RA patients.

## Materials and Methods

### Study population

The medical records of 374 patients diagnosed with RA between January 2011 and June 2012 at Seoul St. Mary’s hospital (the tertiary referral rheumatology center in Korea) were analyzed retrospectively. All patients met the 2010 Rheumatoid arthritis classification criteria [18]. Of the 374 patients, 88 did not have baseline hand X-rays or at least one set of available follow-up radiographs, 85 were lost to follow-up, and seven had seronegative RA. The presentation of rheumatoid factor (RF) and/or anti-citrullinated protein antibodies (ACPA) is known predictive factors for development of erosions and the degree of radiological progression [11]. In the present study, the effect of mechanical stress on radiographic progression was focused and serologic factors were limited. Therefore, the remaining 194 patients were included in the study. All were recently diagnosed with seropositive RA and had both baseline and follow-up hand radiographs. The study was approved by the Institutional Review Board of Seoul St. Mary’s Hospital (KC14RISI0524). Informed consent was waived for this retrospective study and patient information was anonymized and de-identified prior to analysis.

### Radiographic evaluation

Radiographs of both hands (posteroanterior view) were scored using the van der Heijde-modified Sharp Score (HSS) [19]. Briefly, radiological damage in each hand was scored according to the degree of erosions and JSN. In addition, the following hand joints were analyzed separately: the proximal interphalangeal joints (PIPs), the metacarpal joints (MCPs), and the wrist joints. The first interphalangeal joint was included in the PIP joint group and the first carpometacarpal joint was included in the wrist joint group. The sum of the total erosion score for the hands ranged from 0–160 and the total JSN score ranged from 0–120. The total hand HSS ranged from 0–280. Paired hand radiographs were scored by two experienced rheumatologists independently. They were blinded to the patient characteristics, the study aims, and hand dominance. The initial and 2 years follow-up hand radiographs were read in chronological order. To estimate inter-observer reliability, 40 randomly selected films were interpreted twice by each reader at an interval of at least 4 weeks. The Cohen’s κ value for the two readers varied from 0.86 to 0.94. The inter-observer correlation coefficient varied from 0.86 to 0.97. Thus, the average score for the two readers was used for the final analysis. The intraclass correlation coefficients for the two readers ranged from 0.79 to 1 and from 0.83 to 1.

The annual radiographic progression rate (ΔHSS/year) was calculated by dividing the change in hand HSS by the number of years that had elapsed between the baseline and follow-up hand X-rays. A clinically significant change in the total HSS, which includes hands and feet, is 5 [20]; the present study regarded a change in the hands HSS score of 3 as clinically significant. The hand HSS for each incidence of erosion or JSN was categorized as “no progression” (ΔHSS < 1.5) or “progression” (ΔHSS ≥ 1.5/year).

### Potential contributing factors

Factors with the potential to influence joint damage progression were also considered. These included disease activity at baseline (determined by measuring acute phase reactants such as C-reactive protein (CRP) and the erythrocyte sedimentation rate (ESR)), the baseline hand HSS, and the number of disease flare-ups involving the hands (estimated according to the number of ultrasound determined synovitis). The use of methotrexate (MTX), other conventional and biologic disease-modifying anti-rheumatic drugs (DMARDs), and glucocorticoids during the follow-up period was also recorded as dichotomous variables (yes/no) if patients had been taking them for more than 3 months.

Hand dominance was assessed by asking the patient to state which they used predominantly during daily life. The interview was undertaken by nurses in the outpatient clinic who were blinded to the results of the hand X-rays and laboratory findings.

### Bone mineral density in the right and left proximal phalanges

Periarticular and mid-bone bone mineral density (BMD) was measured in the second to the fifth proximal phalanges of both hands to assess the degree of periarticular osteoporosis as previously described [21]. Mid-bone BMD was used as the individual reference value and the ratio of mid-bone-to-periarticular BMD was calculated. These ratios were compared between the right and left hands of 45 RA patients from a different cohort. Overall, 86.3% of Koreans are right-handed and more than 90% of female participants over the age 40 years are right-handed [22]. Therefore, we presumed that the right hand was the dominant hand, as handedness could not be interviewed in those patients.

### Statistical analysis

All statistical analyses were performed using R language ver. 3.01 (R Foundation for Statistical Computing, Vienna, Austria). The HSS for the different joint groups in each hand at baseline and at 2-year follow-up were compared using the Wilcoxon signed rank test. The Wilcoxon signed rank test was also used to compare BMD in the second to the fifth PIPs of right and left hands.

Generalized Estimating Equation (GEE) regression models were used to examine the relationship between the dominant hand joint damage progression (erosions, JSN, and both) and potential risk factors such as handedness during the 2-year follow-up period. Annual changes in the HSS related to erosions, JSN, and both calculated in each hand and combined score changes per year in dominant and non-dominant hand were used as the continuous outcome variable for all analyses. Separate models were used to assess the effect size of independent variables in relation to the HSS changes. Combined GEE models were used to evaluate the contribution of each of the variables associated with radiographic progression (as identified by univariate analysis) in the presence of other dependent variables. When interpreting the GEE analyses, the progression rate (HSS changes per year) of non-dominant hand was considered as the reference. Adjustments were made for possible confounding variables (age, sex, anti-rheumatic drug use, and the initial HSS) associated with radiographic progression. P values < 0.05 were considered statistically significant. The radiographic progression of joint erosion, JSN, or both, was estimated by Kaplan-Meier analysis. Non-progression was examined using the log-rank test.

## Results

### Baseline characteristics

In total, 194 patients (80% females, 88% RF-positive, 92% ACPA-positive) with at least one 2-year hand radiograph were included in the analyses (Table 1). All patients were Korean and 95.4% (n = 185) were right-handed. Most (90.7%, n = 165) were never smokers. The mean age (SE) at RA diagnosis was 50.7 (1.0) years. During follow-up, 75.8% of patients (n = 147) were prescribed glucocorticoids more than 3 months, and 88.7% received MTX (mean dose, 9.7 (0.3) mg per week). Fourteen percent (n = 27) of patients began to use biologics during the 2-year follow-up period. The baseline mean (SE) ESR was 43.2 (2.2) mm/hr and the baseline mean (SE) CRP level was 1.3 (0.2) mg/dL. The mean (SE) follow-up duration was 2.0 (0.1) years, and 54.1% of the patients (n = 105) experienced hand joint flare-up and among them, 80 patients (76.2%) showed dominant hand joint flare-up. The location and number of joint flare up was not different according to the handedness.

**Table 1:** Baseline characteristics of the 194 patients with newly-diagnosed rheumatoid arthritis.

Parameters	N (%)^a^
Age, years	50.7 ± 1.0
Female	155 (79.9)
Dominant hand	
Right	185 (95.4)
Left	9 (4.6)
Documented hand synovitis during follow-up	105 (54.1)
Smoking status at diagnosis	
Non-smoker	165/182 (90.7)
Ex-smoker	6/182 (3.3)
Current smoker	11/182 (6.0)
Rheumatoid factor^b^, IU/mL	174.6 ± 15.4
Anti-citrullinated peptide antibody^c^, U/mL	160.6 ± 8.4
Baseline erythrocyte sediment rate, mm/hr	43.2 ± 2.2
Baseline C-reactive protein^d^, mg/dL	1.3 ± 0.2
Treatment	
Methotrexate use	172 (88.7)
- Methotrexate dose, mg/week	9.7 ± 0.3
Use of other conventional DMARDs	139 (71.6)
Glucocorticoid use	147 (75.8)
Biologics	27 (14.1)
Mean follow-up duration, years	± 0.1
- Patients without joint damage at baseline	39.18)
- Joint damage progression during follow-up	64 (32.99)

DMARDs, disease-modifying anti-rheumatic drugs

^a^ Plus-minus values represent the mean ± SE.

^b^ Reference range, 0–20 IU/mL.

^c^ Reference range, < 7 U/mL.

^d^ Reference range, 0.01–0.47 IU/mL.

### Baseline and follow-up HSS in the dominant and non-dominant hands

**Fig 1:**
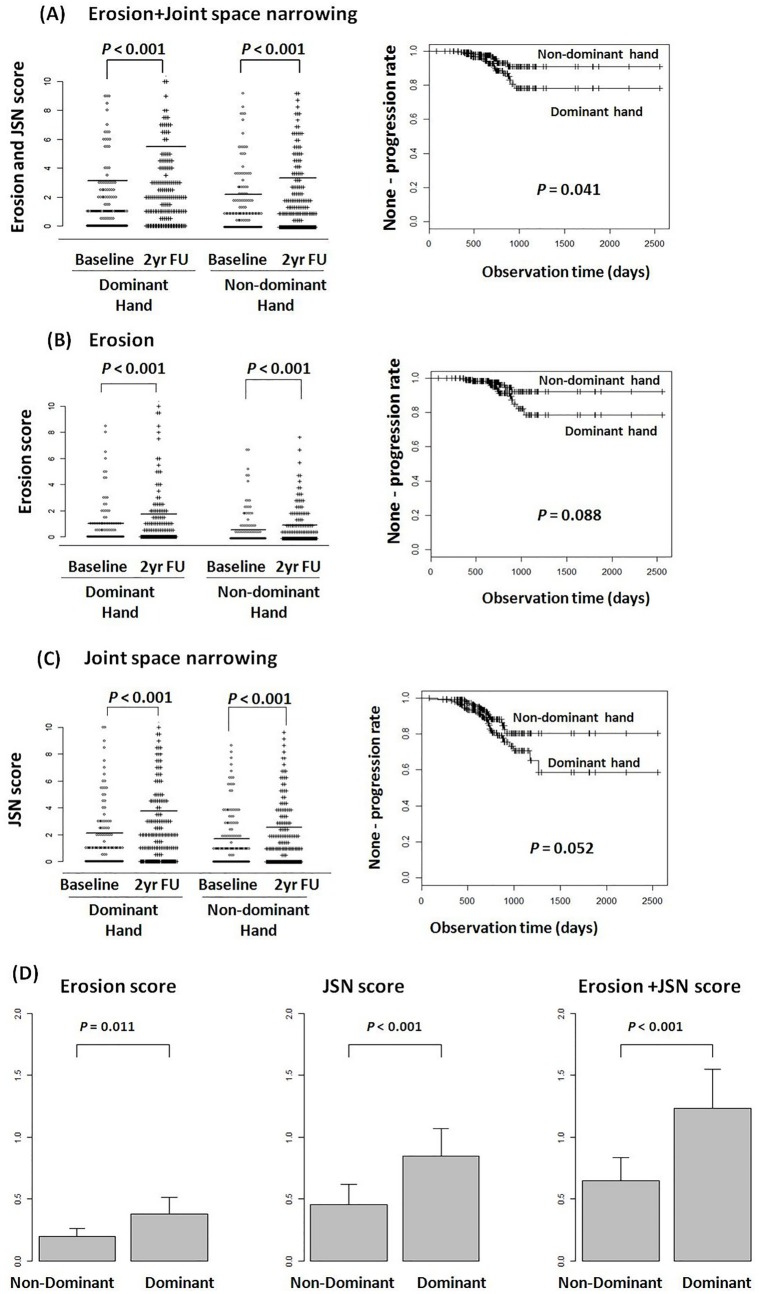
Hand total, erosion and joint space narrowing (JSN) van der Heijde modified Sharp scores (HSS) at baseline and at 2-year follow-up, time-to-radiographic progression and annual progression according to handedness. Radiographic progression was defined as a change in the erosive or JSN HSS ≥ 1.5 and a total HSS ≥ 3 in both hands during the follow-up period. (A) Baseline and follow-up total HSS were significantly higher in the dominant hand than in the non-dominant hand. The Log-rank test identified a significant difference in overall progression between the dominant and non-dominant hand (*P* = 0.041). (B and C) Baseline and follow-up erosion and JSN score were significantly higher in the dominant hand than in the non-dominant hand. The overall erosion and JSN progression rates were 5.7% and 7.5% respectively, during the 2-year follow period. (D) Annual progression of erosion, JSN, and both, were more rapid in the dominant hand.

The baseline HSS for both hands was 0 in 39% (n = 76) of patients. The mean (SE) baseline HSS for the dominant hand was 3.1 (0.5), whereas that for the non-dominant hand was 2.4 (0.4) (Fig 1A). The baseline erosion and JSN scores were also higher in the dominant hand than in the non-dominant hand (Fig 1B and 1C). The HSS for the dominant hand was also higher than that for the non-dominant hand at the 2-year follow-up: 5.5 (0.6) versus 3.7 (0.5), respectively.

Radiographic progression, as defined by increase in the HSS ≥ 3, was observed in 33% of patients (n = 64) during the follow-up period. The mean annual changes in HSS for erosion, JSN, and both, were higher for the dominant hand than for the non-dominant hand (Fig 1D). The HSS for erosions and JSN in the wrist joints were much higher than those for the PIPs and MCPs (Table 2). In the PIPs, JSN progression was greater in the dominant hand than in the non-dominant hand; however, there was no significant difference in progression of erosion in these joints between the dominant and non-dominant hand. Erosion and JSN progression in the MCPs was not significantly different between the dominant and non-dominant hand.

**Table 2:** Annual changes in the van der Heijde modified Sharp score between the dominant and non-dominant hand.

Score	Non-Dominant	Dominant	p-value
	Mean±SE	Mean±SE	
Erosion + JSN	0.65±0.10	1.23±0.16	< 0.001
Erosion	0.20±0.03	0.38±0.07	0.011
PIPs	0.01±0.01	0.04±0.02	0.116
MCPs	0.01±0.01	0.05±0.02	0.072
Wrist	0.16±0.03	0.29±0.06	0.044
JSN	0.46±0.08	0.85±0.11	< 0.001
PIPs	0.08±0.03	0.20±0.04	< 0.001
MCPs	0.06±0.03	0.11±0.03	0.156
Wrist	0.31±0.06	0.54±0.09	< 0.001
Patients without initial radiographic joint damage			
Erosion	0.08±0.03	0.08±0.03	>0.999
JSN	0.25±0.06	0.58±0.11	<0.001
Erosion+JSN	0.33±0.06	0.67±0.12	0.002
Patients with initial radiographic joint damage			
Erosion	0.27±0.05	0.57±0.11	0.008
JSN	0.59±0.13	1.02±0.17	<0.001
Erosion+JSN	0.85±0.15	1.59±0.25	<0.001
Patients with normal CRP			
Erosion	0.16±0.04	0.15±0.04	0.657
JSN	0.35±0.12	0.62±0.10	0.005
Erosion+JSN	0.51±0.13	0.76±0.12	0.016
Patients with elevated CRP			
Erosion	0.23±0.06	0.63±0.13	<0.001
JSN	0.57±0.12	1.10±0.20	<0.001
Erosion+JSN	0.80±0.14	1.74±0.30	<0.001

JSN, joint space narrowing; MCPs, metacarpal joints; PIPs, proximal interphalangeal joints; SE, Standard error

Patients with no initial evidence of radiographic joint damage in either hand yielded an HSS of 0 at baseline and showed an average progression score of 0.9/year, whereas patients with evidence of radiographic joint damage at baseline had a progression score of 2.5/year. For patients with no initial radiographic joint damage, the progression of erosion was not significantly different between the dominant and non-dominant hands, although there was a significant difference in JSN progression between hands. However, for patients with initial radiographic joint damage, both erosion and JSN progressed significantly (*P* = 0.008 and *P* < 0.001, respectively) more quickly in the dominant hand. A similar relationship was found when considering the initial CRP levels: patients with a normal CRP level (reference value, 0.01–0.47 mg/dL) at baseline had an average progression score of 1.2/year while those with abnormal CRP levels at baseline had an average score of 3.0/year. There was no significant difference in erosion between the dominant and non-dominant hand in the normal CRP group; however, JSN increased significantly according to handedness. Finally, progression of both erosion and JSN was more rapid in the dominant hand than in the non-dominant hand of patients with abnormal CRP levels.

### Cumulative incidence of radiographic joint damage progression

Radiographic progression was defined as a change in the erosive or JSN HSS ≥ 1.5 and a total hand HSS ≥ 3 at follow-up. Erosive radiographic progression occurred in 5.7% (n = 22) of patients, JSN progression occurred in 13.4% (n = 52) of patients, and overall progression (both erosion and JSN) occurred in 7.5% of patients (n = 29) during the follow-up period. The dominant hand showed greater total progression than the non-dominant hand (*P* = 0.041) (Fig 1A). However, there was no significant difference in the degree of erosive and JSN progression between hands (*P* = 0.088 and *P* = 0.052, respectively) (Fig 1B and 1C).

### Radiographic progression according to handedness

Single GEE analysis revealed that the annual rate of erosive progression (ΔHSS/year) was associated with abnormal levels of acute phase reactants (ESR and CRP), glucocorticoid use, handedness, and a higher baseline erosive HSS. A high positive ACPA and the use of biologics were also associated with the annual rate of JSN progression. In particular, the use of conventional DMARDs was negatively correlated with the progression of JSN and progression of both erosion and JSN (Table 3). Age, sex, use of biologic DMARDs, and the number of joint flare-up was not associated with radiographic joint damage.

**Table 3:** Demographic and disease factors associated with the rate of annual progression in the dominant and non-dominant hand according to single GEE analysis.

Variable	Erosion		JSN		Erosion + JSN	
	β coefficient	p-Value	β coefficient	p-Value	β coefficient	p-value
Dominant hand	0.18	0.004	0.39	<0.001	0.58	<0.001
Abnormal ESR^a^	0.26	<0.001	0.36	0.009	0.62	<0.001
Abnormal CRP^b^	0.28	0.002	0.35	0.035	0.63	0.005
High-positive ACPA^c^			0.39	0.001	0.50	0.003
Other cDMARDs			-0.50	0.012	-0.58	0.047
Steroid use	0.23	0.002	0.50	<0.001	0.73	<0.001
Baseline HSS	0.08	<0.001	0.05	0.028	0.08	<0.001

ACPA, anti-citrullinated peptide antibodies; cDMARDs, conventional disease-modifying anti-rheumatic drugs; CRP, C-reactive protein; ESR, erythrocyte sediment rate; HSS, van der Heijde modified Sharp score; MTX, methotrexate; RF, rheumatoid factor; JSN, joint space narrowing

^a^ Reference range, < 15 mm/hr.

^b^ Reference range, 0.01–0.47 IU/mL.

^c^ Reference range, < 7 U/mL. A high positive ACPA value > 21 U/mL (three times the upper normal limit).

Multiple GEE analyses (including all associated variables identified by the single GEE model) showed that the dominant hand, the HSS for erosion, JSN, and both at baseline, and abnormal CRP levels were independently associated with annual radiographic progression of erosion, JSN, and both. The use of conventional DMARDs was negatively correlated with JSN (Table 4).

**Table 4:** Factors associated with the rate of annual progression in the dominant and non-dominant hand according to multivariate GEE analysis.

Variable	Erosion		JSN		Erosion and JSN	
	β coefficient	p-Value	β coefficient	p-Value	β coefficient	p-value
Dominant hand	0.16	0.032	0.38	0.006	0.53	0.003
Abnormal CRP^a^	0.25	0.001	0.29	0.044	0.52	0.006
Baseline HSS	0.07	<0.001	0.05	0.033	0.08	<0.001
Other cDMARDs use			-0.51	0.002	-0.59	0.010

cDMARDs, conventional disease-modifying anti-rheumatic drugs; CRP, C-reactive protein; HSS, van der Heijde modified Sharp score; JSN, joint space narrowing.

^a^Reference range, 0.01–0.47 IU/mL

### BMD in both hands

BMD was measured in the hands of 45 patients in another cohort and the mid-to-periarticular BMD ratio for the second to the fifth proximal phalanges in the right hand was compared with that in the left. There was no significant difference in BMD in any of the fingers in the right and left hands (Table 5).

**Table 5:** Comparison of the mean mid-bone-to-periarticular BMD ratio in the right and left hands.

	Right mid-bone-to-periarticular BMD ratio (IQR)	Left mid-bone-to-periarticular BMD ratio (IQR)	p-value
2^nd^ PIP	0.52 (0.43–0.63)	0.51 (0.43–0.61)	0.488
3^rd^ PIP	0.51 (0.45–0.61)	0.53 (0.46–0.57)	0.705
4^th^ PIP	0.49 (0.42–0.55)	0.47 (0.42–0.54)	0.664
5th PIP	0.50 (0.45–0.61)	0.49 (0.42–0.60)	0.672
mean	0.51 (0.47–0.59)	0.50 (0.45–0.56)	0.538

BMD, bone mineral density; IQR, interquartile range; PIPs, proximal interphalangeal joints.

## Discussion

The aim of the present study was to examine the association between handedness and radiographic structural damage in patients with RA. The results showed that the rate of progression of radiographic joint damage was worse in the dominant hand than in the non-dominant hand. However, although erosion and JSN in the wrist joints of the dominant hand progressed more quickly than in the non-dominant hand, there was no significant difference between the rate of erosion in the PIP joints in the two hands. By contrast, JSN in the PIPs of the dominant hand progressed more rapidly than that in the non-dominant hand. This discrepancy in the rate of radiographic progression between the dominant and non-dominant hands was not observed for the MCPs. Previous studies show that the PIPs experience greater mechanical stress than the MCPs during common daily activities [23], and that the radiographic OA score is higher for PIPs than for MCPs [24]. Thus, we propose that the differences in the rate of radiographic progression reported herein are due to increased mechanical stress on the PIPs. These findings are different from those of Knevel *et al*. [25], who reported that both radiographic damage at a specific time point and the rate of radiographic progression in the hands are highly correlated. Interestingly, they also found that the correlation between the right and left hands was weaker at baseline than at follow-up, especially in terms of the total JSN score and in the early RA cohort.

Mechanical stress at a joint surface damages the joint tissue, resulting in OA [26]. Therefore, radiographic progression is associated with JSN progression due to mechanical stress. However, hand OA is a slowly progressive disorder; indeed, the OA progression rate is 1.1/6 years according to the OARSI atlas (range 0 to 96) [27]. Compared with this, RA progresses at a much faster rate. Therefore, mechanical stress might play a role in radiographic progression in addition to the degenerative changes associated with RA. In addition, age influences the progression of OA; however, we did not find that erosion and JSN scores differed according to age in the RA patients examined in the present study.

We did observe an association between baseline levels of systemic inflammation (according to CRP levels) and asymmetrical erosive progression over the 2 year follow-up period. Erosive progression in patients with normal CRP levels at baseline did not differ according to handedness; however, the rate of JSN progression in the dominant hand was greater than that in the non-dominant hand. By contrast, greater HSS changes, and clear differences in both erosion and JSN between the dominant and non-dominant hands, were observed in patients with abnormal CRP levels at baseline. Thus, these results support the notion that mechanical stress plays a role in joint erosion in RA, particularly in individuals with an active inflammatory condition. The use of conventional DMARDs (but not biologics) was negatively correlated with the annual rate of radiographic progression. Most of the patients in the present study were taking conventional DMARDs in combination with MTX. Korean national insurance guidelines stipulate that biologics should only be prescribed when a patient is refractory to at least two conventional DMARDs over a period of 6 months. Because the patients in the present study were newly diagnosed with RA, conventional DMARDs were prescribed to control active inflammatory disease. Thus, our results imply that it is important to control active inflammatory disease if one is to reduce radiographic progression. These findings correspond well with those of the earlier studies which reported that combination DMARDs significantly reduced radiographic progression [28, 29].

Patients with recent onset RA or individuals at risk of RA show a high prevalence of localized cortical erosion [30] and osteopenia [21, 31]. However, we found no difference in BMD between the dominant and non-dominant hands in the present study. Periarticular demineralization is the direct result of an inflammatory process that is triggered by the release of local inflammatory mediators. Thus, we suppose that inflammation accelerates cortical thinning or decreases bone density in both hands to a similar extent.

Taken together, the data presented herein suggest that mechanical stress in the dominant hand may accelerate progression toward cortical breaks and JSN in the context of local inflammatory reactions. Therefore, we suggested that the use of wrist splints or partial finger casts may reduce radiographic joint damage in patients with RA.

A major limitation of the present study is its retrospective nature. This means that we were unable to measure the forces applied to the joint surfaces. Neither did we know the occupation of the patients nor the degree of daily hand use. In addition, the study may be subject to selection bias because the hospital is a tertiary referral center and a large number of patients were excluded due to lack of data.

A second limitation is that the radiologists were not blinded to sequence of x-rays. Although handedness and aim of study were blinded to the readers, they could assume right handed. There were only nine patients who were left handed; four patients showed more radiographic progression in left hands, three patients were not progressed, and two patients showed more radiographic progression in right hands. These two patients who showed higher baseline HSS on right hand.

A third limitation is that we used plain radiographs to assess hand joint damage. The degree of JSN might be influenced by the position of the joint in the plain radiograph and (possibly) by the amount of joint swelling [32]. MRI and ultrasound are playing increasing roles in the diagnosis of early RA; therefore, further studies should be conducted using these modalities.

A fourth limitation is that we could not compare hand radiographs and BMD measurements in the same patient. In addition, we did not know the handedness of the patients in whom BMD was measured. Because BMD was measured in only 45 patients, we cannot assume that the right hand was the dominant hand.

However, the study has some strengths. First, as the non-dominant hand was used for reference in each individual, all other risk factors for radiographic joint damage (including treatment) were relatively well controlled. Second, we examined erosions and JSN (and both) in separate joint groups.

## Conclusions

Higher erosion and JSN scores were observed in the dominant hand of early RA patients during the 2-year follow-up period of this study. The annual rate of radiographic progression was also higher in the dominant hand. However, there was no significant difference in BMD between the dominant and non-dominant hands. These findings suggest that mechanical stress may accelerate radiographic joint damage in patients with active inflammatory RA. Further research on the relationship between changes in inflammatory cytokine levels and mechanical stress is needed to confirm and extend these findings.

## Supporting Information

S1 Table Baseline and follow up (median 4 years) joint space narrowing score according to the OARSI atlas in 30 Patients with hand osteoarthritis. (DOCX)

## References

[1] Arnett FC, Edworthy SM, Bloch DA, McShane DJ, Fries JF, Cooper NS, et al The American Rheumatism Association 1987 revised criteria for the classification of rheumatoid arthritis. Arthritis Rheum. 1988;31:315–324. 10.1002/art.1780310302 3358796

[2] van der Heijde DM, van Leeuwen MA, van Riel PL, Koster AM, van 't Hof MA, van Rijswijk MH, et al Biannual radiographic assessments of hands and feet in a three-year prospective followup of patients with early rheumatoid arthritis. Arthritis Rheum. 1992;35:26–34. 1731813 10.1002/art.1780350105

[3] Wilder FV, Barrett JP, Farina EJ. Joint-specific prevalence of osteoarthritis of the hand. Osteoarthritis and Cartilage. 2006;14:953–957. doi: 10.1016/j.joca.2006.04.013 16759885

[4] Acheson RM, Chan YK, Clemett AR. New Haven survey of joint diseases. XII. Distribution and symptoms of osteoarthrosis in the hands with reference to handedness. Ann Rheum Dis. 1970;29:275–286. 5432594 10.1136/ard.29.3.275 PMC1031263

[5] Brandt KD, Dieppe P, Radin EL. Etiopathogenesis of osteoarthritis. Rheum Dis Clin North Am. 2008;34:531–559. 10.1016/j.rdc.2008.05.011 18687271

[6] Nakamura R, Ono Y, Horii E, Tsunoda K, Takeuchi Y. The aetiological significance of work-load in the development of osteoarthritis of the distal interphalangeal joint. J Hand Surg Br. 1993;18:540–542. 8409676 10.1016/0266-7681(93)90167-e

[7] Bergenudd H, Lindgarde F, Nilsson B. Prevalence and coincidence of degenerative changes of the hands and feet in middle age and their relationship to occupational work load, intelligence, and social background. Clin Orthop Relat Res. 1989:306–310. 2912632

[8] Bland JH, Eddy WM. Hemiplegia and rheumatoid hemiarthritis. Arthritis Rheum. 1968;11:72–80. 10.1002/art.1780110110 5643259

[9] Thompson M, Bywaters EG. Unilateral rheumatoid arthritis following hemiplegia. Ann Rheum Dis. 1962;21:370–377. 10.1136/ard.21.4.370 13981183 PMC1007306

[10] Glick EN. Asymmetrical rheumatoid arthritis after poliomyelitis. Br Med J. 1967;3:26–28. 6027379 10.1136/bmj.3.5556.26 PMC1845190

[11] Machold KP, Stamm TA, Nell VP, Pflugbeil S, Aletaha D, Steiner G, et al Very recent onset rheumatoid arthritis: clinical and serological patient characteristics associated with radiographic progression over the first years of disease. Rheumatology (Oxford). 2007;46:342–349. 10.1093/rheumatology/kel237 16899498

[12] Fex E, Jonsson K, Johnson U, Eberhardt K. Development of radiographic damage during the first 5–6 yr of rheumatoid arthritis. A prospective follow-up study of a Swedish cohort. Br J Rheumatol. 1996;35:1106–1115. 10.1093/rheumatology/35.11.1106 8948297

[13] Scott DL, Pugner K, Kaarela K, Doyle DV, Woolf A, Holmes J, et al The links between joint damage and disability in rheumatoid arthritis. Rheumatology (Oxford). 2000;39:122–132. 10.1093/rheumatology/39.2.122 10725061

[14] Ødegård S, Landewé R, van der Heijde D, Kvien TK, Mowinckel P, Uhlig T. Association of early radiographic damage with impaired physical function in rheumatoid arthritis: a ten-year, longitudinal observational study in 238 patients. Arthritis Rheum. 2006;54:68–75. 10.1002/art.21548 16385497

[15] Graudal N, Tarp U, Jurik AG, Galloe AM, Garred P, Milman N, et al Inflammatory patterns in rheumatoid arthritis estimated by the number of swollen and tender joints, the erythrocyte sedimentation rate, and hemoglobin: longterm course and association to radiographic progression. J Rheumatol. 2000;27:47–57. 10648017

[16] Devlin J, Lilley J, Gough A, Huissoon A, Holder R, Reece R, et al Clinical associations of dual-energy X-ray absorptiometry measurement of hand bone mass in rheumatoid arthritis. Br J Rheumatol. 1996;35:1256–1262. 9010053 10.1093/rheumatology/35.12.1256

[17] Berglin E, Lorentzon R, Nordmark L, Nilsson-Sojka B, Rantapaa Dahlqvist S. Predictors of radiological progression and changes in hand bone density in early rheumatoid arthritis. Rheumatology (Oxford). 2003;42:268–275. 12595621 10.1093/rheumatology/keg077

[18] Aletaha D, Neogi T, Silman AJ, Funovits J, Felson DT, Bingham CO 3rd, et al 2010 Rheumatoid arthritis classification criteria: An American College of Rheumatology/European League Against Rheumatism collaborative initiative. Arthritis & Rheumatism. 2010;62:2569–2581. 10.1002/art.27584 20872595

[19] van der Heijde D. How to read radiographs according to the Sharp/van der Heijde method. J Rheumatol. 2000;27:261–263. 10648051

[20] Bruynesteyn K, van der Heijde D, Boers M, Saudan A, Peloso P, Paulus H, et al Determination of the minimal clinically important difference in rheumatoid arthritis joint damage of the Sharp/van der Heijde and Larsen/Scott scoring methods by clinical experts and comparison with the smallest detectable difference. Arthritis Rheum. 2002;46:913–920. 10.1002/art.10190 11953967

[21] Moon SJ, Ahn IE, Kwok SK, Park KS, Min JK, Park SH, et al Periarticular osteoporosis is a prominent feature in early rheumatoid arthritis: estimation using shaft to periarticular bone mineral density ratio. J Korean Med Sci. 2013;28:287–294. 10.3346/jkms.2013.28.2.287 23399828 PMC3565142

[22] Jung HS, Jung HS. Hand dominance and hand use behaviour reported in a survey of 2437 Koreans. Ergonomics. 2009;52:1362–1371. 10.1080/00140130903067805 19851904

[23] Butz KD, Merrell G, Nauman EA. A biomechanical analysis of finger joint forces and stresses developed during common daily activities. Comput Methods Biomech Biomed Engin. 2012;15:131–140. 10.1080/10255842.2010.517525 21711164

[24] Caspi D, Flusser G, Farber I, Ribak J, Leibovitz A, Habot B, et al Clinical, radiologic, demographic, and occupational aspects of hand osteoarthritis in the elderly. Semin Arthritis Rheum. 2001;30:321–331. 10.1053/sarh.2001.19957 11303305

[25] Knevel R, Kwok KY, de Rooy DP, Posthumus MD, Huizinga TW, Brouwer E, et al Evaluating joint destruction in rheumatoid arthritis: is it necessary to radiograph both hands and feet? Ann Rheum Dis. 2013;72:345–349. 10.1136/annrheumdis-2012-201391 22580587

[26] Visser AW, de Mutsert R, le Cessie S, den Heijer M, Rosendaal FR, Kloppenburg M. The relative contribution of mechanical stress and systemic processes in different types of osteoarthritis: the NEO study. Ann Rheum Dis. 2014 5 20 10.1136/annrheumdis-2013-205012 24845389

[27] Bijsterbosch J, Watt I, Meulenbelt I, Rosendaal FR, Huizinga TW, Kloppenburg M. Clinical and radiographic disease course of hand osteoarthritis and determinants of outcome after 6 years. Ann Rheum Dis. 2011;70:68–73. 10.1136/ard.2010.133017 20736393

[28] Graudal N, Jurgens G. Similar effects of disease-modifying antirheumatic drugs, glucocorticoids, and biologic agents on radiographic progression in rheumatoid arthritis: meta-analysis of 70 randomized placebo-controlled or drug-controlled studies, including 112 comparisons. Arthritis Rheum. 2010;62:2852–2863. 10.1002/art.27592 20560138

[29] Graudal N, Hubeck-Graudal T, Tarp S, Christensen R, Jurgens G. Effect of combination therapy on joint destruction in rheumatoid arthritis: a network meta-analysis of randomized controlled trials. PLoS One. 2014;9:e106408 10.1371/journal.pone.0106408 25244021 PMC4171366

[30] Funck-Brentano T, Etchepare F, Joulin SJ, Gandjbakch F, Pensec VD, Cyteval C, et al Benefits of ultrasonography in the management of early arthritis: a cross-sectional study of baseline data from the ESPOIR cohort. Rheumatology (Oxford). 2009;48:1515–1519. 10.1093/rheumatology/kep279 19755507

[31] Güler-Yüksel M, Allaart CF, Goekoop-Ruiterman YP, de Vries-Bouwstra JK, van Groenendael JH, Mallee C, et al Changes in hand and generalised bone mineral density in patients with recent-onset rheumatoid arthritis. Ann Rheum Dis. 2009;68:330–336. 10.1136/ard.2007.086348 18375540

[32] van der Heijde D. Erosions versus joint space narrowing in rheumatoid arthritis: what do we know? Ann Rheum Dis. 2011;70(Suppl 1):i116–118. 10.1136/ard.2010.140525 21339214

