# Supplementary material for: Radiographic Structural Damage Is Worse in the Dominant than the Non-Dominant Hand in Individuals with Early Rheumatoid Arthritis
Source: PLoS One. 2015 Aug 6;10(8):e0135409. doi: 10.1371/journal.pone.0135409 (PMC4527732; doi:10.1371/journal.pone.0135409)
Supplement: S1 Table — (DOCX) [file pone.0135409.s001.docx]

**S1 Table. Baseline and follow up**^a^ **joint space narrowing score on hands radiographs according to the OARSI atlas in 30 Patients**^b^ **with hand osteoarthritis**

|  | Right hand | Left hand | p-value |
| --- | --- | --- | --- |
| OARSI JSN score (initial) |  |  |  |
| DIPs | 2 (0–4) | 0 (0–3) | 0.004 |
| PIPs | 0 (0–1) | 0 (0–0) | 0.132 |
| wrist | 0 (0–1) | 0 (0–1) | 0.782 |
| Total score | 3 (1–4.5) | 1.5 (0–3.3) | 0.001 |
| OARSI JSN score (follow- up) |  |  |  |
| DIPs | 3 (0.5–5.3) | 2 (0–4) | 0.009 |
| PIPs | 0 (0–1) | 0 (0–0.3) | 0.109 |
| wrist | 0 (0–1) | 0 (0–1) | >0.999 |
| Total score | 4 (1.8–7.3) | 3 (0–5.5) | 0.003 |
| Annual progression |  |  |  |
| DIPs | 0 (0–0.36) | 0.12 (0–0.28) | 0.913 |
| PIPs | 0 (0–0) | 0 (0–0) | 0.484 |
| wrist | 0 (0–0) | 0 (0–0) | 0.285 |
| Total score | 0.13 (0–0.42) | 0.12 (0.0–0.38) | 0.821 |

Scores are presented as median (interqurtile) and p-values are calculated with the Wilcoxon singed rank test.

^a^ The median time interval between initial and follow-up radiographs was 4.0 (2.8–6.0) years.

^b^ The median age (IQR) of these 30 patients was 64 (56–68).

JSN, joint space narrowing; MCPs, metacarpal joints; OARSI, Osteoarthritis Research Society International; PIPs, proximal interphalangeal joints
